# Manure-Amended One-Year-Reclamation Promoted Soil Bacterial Phylotypic and Phenotypic Shifts in a Typical Coal-Mining Area

**DOI:** 10.3390/microorganisms13040699

**Published:** 2025-03-21

**Authors:** Hongjuan Zhang, Yanmeng Shang, Shuning Bai, Meihua Fan, Xiaolong Sui, Huisheng Meng, Xianjun Hao, Xiangying Wang, Yulin Liu, Yi Li, Jianping Hong, Jie Zhang

**Affiliations:** 1 College of Resources and Environment, Shanxi Agricultural University, Jinzhong 030801, China; zhanghongjuan@sxau.edu.cn (H.Z.); shangyanmeng163@163.com (Y.S.); baishuningbsn@163.com (S.B.); 18335486929@163.com (M.F.); asdfsxl@gmail.com (X.S.); huishengmeng@126.com (H.M.); haoxianjun660@126.com (X.H.); liuyulin191@163.com (Y.L.); hongjpsx@163.com (J.H.); 2 Soil Health Laboratory in Shanxi Province, Shanxi Agricultural University, Taiyuan 030031, China; 3 National Experimental Teaching Demonstration Center for Agricultural Resources and Environment, Shanxi Agricultural University, Jinzhong 030801, China; 4 College of Resources and Environmental Sciences, Nanjing Agricultural University, Nanjing 210095, China; 5 College of Life Science, Shanxi Agricultural University, Jinzhong 030801, China; wxyzlb@163.com (X.W.); liyi@sxau.edu.cn (Y.L.)

**Keywords:** bacterial diversity, bacterial phenotype, coal-mining reclamation, fertilization practices, manure

## Abstract

The initial variations in soil bacteria at the very beginning of reclamation still remains unclear. This study investigates the impact on bacterial communities of eight different treatments, including uncultivated land, unfertilized cultivation, chemical fertilizer, chemical fertilizer + bacterial fertilizer, manure, manure + bacterial fertilizer, manure + chemical fertilizer, and manure + chemical fertilizer + bacterial fertilizer, during the short-term reclamation of coal-mining soils. The results showed that total nitrogen, available phosphorus, soil organic carbon, microbial biomass carbon, and alkaline phosphatase activity were significantly increased in all fertilization treatments compared to uncultivated land (*p* < 0.05). All fertilization treatments other than chemical fertilizer harbored significantly higher activities of urease, catalase, and invertase than unfertilized cultivation (*p* < 0.05). The bacterial communities structures in manure-amended treatments significantly differed in uncultivated land and unfertilized cultivation and were phylotypically shifted from oligotrophic to *Actinobacteria*-dominant copiotrophic traits, accompanied with phenotypic succession of the enriching characteristics of Gram-positive, biofilms formation, and stress tolerance. The co-occurrence network in manure-amended treatments harbored a simple co-occurrence network, indicating more productive soils than in no-manure treatments. Manure amendment, total nitrogen, microbial biomass carbon, invertase, catalase, and soil moisture were the key driving factors. Our study underscores the bacterial initialization characteristics promoted by manure at the very beginning of coal-mining reclamation.

## 1. Introduction

Coal mining is an essential industrial activity that supports economic development and energy production worldwide. However, it often comes at a significant environmental cost. The disruption of soil profiles, the alteration of landscape topography, and the generation of waste materials in coal-mining operations lead to severe ecological disturbances, including the loss of soil organic matter, reduced fertility, and diminished biodiversity [1,2,3]. Moreover, substantial land areas degraded by coal mining exhibit an extensive overlap with croplands, posing substantial threats to food security [4,5]. These consequences pose substantial challenges for post-mining land management and highlight the need for effective reclamation strategies to mitigate the adverse impacts and facilitate ecosystem recovery. Fertilization with revegetation restoration is a highly effective approach to enhancing soil fertility and restoring degraded soils in mining areas [6]. By adding organic or mineral fertilizers, key nutrients such as nitrogen, phosphorus, and potassium are replenished, which directly improves soil health and productivity [7]. This practice not only boosts crop yields but also stimulates microbial activity, fostering a more balanced and sustainable soil ecosystem [8,9].

Soil microorganisms, particularly bacteria, play an integral role in maintaining soil health through processes such as decomposition, nutrient cycling, and soil structure stabilization [10]. The diversity and activity of these microorganisms are intricately connected to soil fertility and ecosystem functionality. Consequently, the restoration of soil microbial communities is a critical component of successful land reclamation efforts following coal-mining activities. Previous studies have examined bacterial community variations in coal-mining-impacted soils and demonstrated that fertilization is an effective strategy for promoting bacterial diversity recovery and improving soil properties [6,11,12,13]. Various types of fertilizers, including inorganic, organic, organic–inorganic blends, and coal-derived compound fertilizers, have been tested for their effectiveness [14]. Beyond bulk soil improvements, these fertilization practices also enhance bacterial diversity and increase the complexity of bacterial ecological networks in rhizosphere soils [15,16].

However, existing studies primarily focus on general changes in bacterial biomass, diversity, and community structure after several years of fertilization and reclamation efforts as it has been reported that the most important phase of microbial community recovery occurs between 5 and 14 years after reclamation [6,11,12,13,17]. Notably, important details remain unclear regarding the specific phylotypic and phenotypic biomarkers that are significantly enriched at the very beginning of the reclamation. These key microbial taxa or phenotypes play a critical role in shaping microbial metabolic properties and community structures [18]. Understanding this phenomenon is essential for explaining how fertilization can reduce recovery times, particularly when reclamation duration is the primary driver of bacterial community restoration in coal-mining-impacted soils [6]. This knowledge gap currently limits our ability to develop optimized reclamation strategies that effectively leverage the potential of soil microorganisms for ecosystem restoration. Addressing these gaps will be crucial for advancing sustainable land management practices in post-coal-mining landscapes.

Therefore, in this study, we aim to address knowledge gap relating to the bacterial phylotypic and phenotypic shifts at the very beginning of reclamation under different fertilization regimes. The study is based on a long-term in situ-positioning reclamation experiment for a coal-mining area in Shanxi Province, the largest coal-producing region in China. Quantitative PCR and high-throughput sequencing of the bacterial 16S rRNA gene were used to determine the soil bacterial biomass and diversity after one-year reclamation under eight regimes. We hypothesized that the application of different fertilizers would promote the recovery of soil bacterial communities by enriching specific phylotypes and phenotypes, thereby enhancing soil health and fostering ecosystem regeneration. Through this targeted experiment, we sought to identify key bacterial pioneer species that initiate the recovery of soil microbial ecology, as well as the driving factors, and provide valuable guidance for developing sustainable reclamation practices tailored to coal-mined landscapes.

## 2. Materials and Methods

### 2.1. Experimental Design and Sampling

The study site is located in Hougou Village, Wujinshan Town, Yuci District, Jinzhong City, Shanxi Province (37°50′19.97″ N, 112°48′21.58″ E), which is on the Loess Plateau in China (Figure S1). The area harbors a temperate continental monsoon climate, with an average annual temperature of 9–10 °C, an average annual precipitation of 462 mm, and a frost-free period of 175 days. In 2019, raw calcareous cinnamon soil (Calciustepts) was taken from the nearby mountains to cover the gangue landfill, with an average thickness of 1 m after mechanical leveling. The initial physicochemical properties of the raw soil were described previously [19].

Thereafter, the leveled land has been further biologically reclaimed since 2020 for study. This experiment used a randomized block complete design with eight different treatments and three replications. Each replicate was a plot of 10 m × 5 m = 50 m^2^. The treatments included UL (uncultivated land), CK (maize cultivation without fertilization), NPK (maize cultivation with chemical fertilizer), NPKB (maize cultivation with co-application of chemical fertilizer and bacterial fertilizer), M (maize cultivation with manure), MB (maize cultivation with co-application of manure and bacterial fertilizer), MNPK (maize cultivation with co-application of manure and chemical fertilizer), and MNPKB (maize cultivation with co-application of manure, chemical fertilizer and bacterial fertilizer). The chemical fertilizer was composed of urea (N, 46%), calcium superphosphate (P_2_O_5_, 12%), and potassium chloride (K_2_O, 60%). The manure was chicken manure compost with 27.8% organic matter, 1.68% nitrogen, 1.54% P_2_O_5_, and 0.82% K_2_O. The bacterial fertilizer was two phosphate-solubilizing bacteria, *Pseudomonas fluorescein* strain W134 and W137, isolated from calcareous reclaimed soil [20,21]. The fertilization amounts for each treatment are shown in Table S1. Maize (*Zea mays* Linn.) was sown in late April with a planting density of 60,000/hectare.

Twenty-four topsoil samples (0–20 cm) were collected using the five-point mixed sampling method from each plot before maize harvest on 26 September 2020. The samples were transported to the lab in an icebox. In the lab, plant residues and detritus were firstly removed from the soil samples, each of which was then divided into two parts. The two subsamples were, respectively, stored at 4 °C for biophysicochemical analyses and −80 °C for microbial analysis.

### 2.2. Soil Biophysicochemical Properties Analyses

Soil physicochemical properties were analyzed according to the methods of Bao [22]. Briefly, soil moisture (SM) was measured using the oven-drying method. Total nitrogen (TN) and alkaline nitrogen (AN) were determined using the semimicro-Kjeldahl method and the alkali N-proliferation method, respectively. Total phosphorus (TP) and available phosphorus (AP) were measured via the alkali fusion-Mo-Sb anti-spectrophotometric method and the sodium-bicarbonate-extraction colorimetric method. Total potassium (TK) and available potassium (AK) were, respectively, extracted using sodium hydroxide and ammonium acetate, and measured by flame photometry. Soil organic carbon (SOC) was determined using the potassium dichromate volumetric method. Soil microbial biomass carbon (MBC) was measured by the chloroform-fumigation extraction method. Soil dissolved organic carbon (DOC) and easily oxidizable organic carbon (EOC) were determined by the distillation water extraction method and potassium permanganate oxidation method. Soil particulate organic carbon (POC) was measured according to Cambardella and Elliott [23].

Soil alkaline phosphatase (ALP) and catalase (CAT) activities were, respectively, determined by the p-nitrophenyl phosphate colorimetric method [24] and the potassium permanganate titration method [25]. Soil invertase (INV) and urease (URE) activities were, respectively, determined by the 3, 5-dinitrosalicylic acid colorimetric method [26] and the indophenol blue colorimetric method [27].

### 2.3. Soil Bacterial 16S rRNA Gene Quantification and Sequencing

Total DNA was extracted from 0.25 g each soil sample using a PowerSoil DNA Isolation Kit (QIAGEN, Hilden, Germany) according to the manufacturer’s instruction. Absolute quantitative PCR (qPCR) was applied to determine the soil bacterial abundance with the 16S rRNA gene-targeted primer set (338F: 5′-CCTACGGGAGGCAGCAG-3′, 518R: 5′-ATTACCGCGGCTGCTGG-3′) using a QuantGene 9600 real-time PCR system (Bioer Technology, Hangzhou, China) [28]. The reaction was carried out in a final volume of 20 μL, including 10 μL of 2× SYBR^®^ Premix (Biomed, Beijing, China), 0.8 μL of each primer, 1.4 μL of DNA template, and 7 μL of ddH_2_O. The reaction conditions were initial denaturation at 95 °C for 10 min, followed by 40 cycles of denaturation at 95 °C for 30 s, and annealing at 60 °C for 1 min. Ultimately, amplification specificity was verified by the melting curve. The qPCR efficiency was 98% (R^2^ = 0.998).

Soil bacterial sequencing was performed on an Illumina Miseq PE300 platform by Shanghai Majorbio Bio-pharm Technology Co., Ltd. (Shanghai, China) using the primer sets 341F (5′-CCTACGGGNGGCWGCAG-3′) and 806R (5′-GGACTACHVGGGTATCTAA T-3′) targeting the V3-V4 region of the bacterial 16S rRNA gene [12].

### 2.4. Data Processing and Statistical Analyses

The bacterial 16S rRNA gene sequencing data were analyzed on the Majorbio Cloud platform (www.majorbio.com, accessed on 15 October 2022). Briefly, the paired-end raw reads were demultiplexed to individual samples and quality-filtered by FASTP (V0.19.6), and the merged by FLASH (v1.2.11). Then, the high-quality sequences with 97% similarity were clustered into the same OTU (operational taxonomic unit) using UPARSE (v11). The most abundant sequence for each OTU was selected as a representative sequence. Non-bacterial 16S rRNA gene reads were removed from the OTU table. To minimize the effects of sequencing depth on alpha and beta diversity estimation, the sequences were randomly subsampled down to 33,673, which was the lowest number of sequences among all samples.

Taxonomic classification of the representative sequences was performed using the RDP Classifier (v2.2) based on the SILVA database (v138), with a confidence threshold value of 0.8. Rarefaction curves and alpha diversity indices, including the Sobs richness index, Shannon–Weaver diversity index, and Pielou evenness index, were calculated with Mothur (v1.30.2). The differences in bacterial community structure among different samples at genera level were determined, respectively, by non-metric multidimensional scaling (NMDS) and a biclustering heatmap using Vegan package (v2.5.3) and ComplexHeatmap package (v2.13.1). The linear discriminant analysis (LDA) effect size (LEfSe) was performed to identify the significantly enriched bacterial taxa among the different groups (LDA score > 4, *p* < 0.05). The BugBase algorithm was used to conduct bacterial phenotype prediction based on taxonomic classification according to the GreenGenes database (V135). The networks were constructed to investigate the bacterial community relationships across the samples based on statistically robust Spearman’s correlation events (|*R*| > 0.60, *p* < 0.01).

The distance-based redundancy analysis (db-RDA) was performed using Vegan package (v2.5.3) to investigate bacterial community structure differences and the driving factors among reclamation treatments. Before this, forward selection was performed using VIF (variance inflation factor), and environmental variables with a VIF > 5 were eliminated before db-RDA [19]. Structural equation modeling (SEM) was constructed to further elucidate the relationships between a bacterial community and the environmental variables.

All statistical analyses and multivariate analyses, and relevant graph plotting, were conducted in R environment (v4.0.3). The sequencing raw reads were submitted to the SRA (Sequence Read Archive) database of the NCBI with the accession number PRJNA948914.

## 3. Results

### 3.1. Soil Biophysicochemical Properties

The soil biophysicochemical properties of different treatments are listed in Table 1. As for basic properties, compared with UL, TN was significantly increased in all reclamation treatments other than CK, with the maximum in MB (*p* < 0.05); AP was significantly increased in all reclamation treatments (*p* < 0.05), with the maximum in MNPK; however, TK was significantly decreased in all reclamation treatments other than MB (*p* < 0.05), with the minimum in NPK. Compared with CK, AN content was significantly decreased in NPKB, MNPK, and MNPKB (*p* < 0.05).

As for the soil carbon fractions, compared with UL and CK, the SOC was significantly increased under all fertilization treatments (*p* < 0.05), with the maximum in MB. Otherwise, the SOC content in manure treatments (M, MB, MNPK, and MNPKB) was significantly higher than treatments with chemical fertilizer (NPK and NPKB) (*p* < 0.05). The content of the MBC in all reclamation treatments was significantly higher than in UL, and significantly higher in all fertilization treatments than in CK (*p* < 0.05), with the maximum in MNPKB. The bacterial fertilizer co-application treatments (NPKB, MB, and MNPKB) exhibited elevated the SOC and MBC contents compared to their respective non-co-application counterparts (NPK, M, and MNPK). However, other soil organic carbon fractions (EOC, DOC, and POC) had no significant differences among the treatments (*p* > 0.05).

As for soil enzymatic activity, all fertilization treatments significantly increased the ALP activity compared with in UL and CK (*p* < 0.05), with the maximum in MB. All fertilization treatments other than NPK harbored significantly higher activities of URE, CAT and INV than in CK (*p* < 0.05). Furthermore, in all bacterial fertilizer co-application treatments (NPKB, MB, and MNPKB), the activities of the four enzymes were significantly higher compared to their corresponding non-co-application counterparts (NPK, M, and MNPK).

### 3.2. Bacterial Biomass and Alpha Diversity

As Figure S2 lists, the soil bacterial biomass based on 16S rRNA gene abundance significantly differed among different treatments. Compared with UL and CK, all fertilization treatments other than NPKB, especially the manure treatments (M, MB, MNPK, and MNPKB), increased bacterial biomass, with a significant (*p* < 0.05) and maximum increase in MB (1.51 × 10^9^ copies/g dry soil).

Based on high-throughput sequencing, in total, 4,004,135 raw reads were obtained, and 1,506,238 high-quality reads were left after quality control. High-quality reads from each sample were then randomly subsampled to 33,673 reads. Subsequently, they were classified into 39 phyla, 129 classes, 248 orders, 413 families, 642 genera, and 3705 OTUs.

The soil bacterial α-diversity indexes under different fertilization treatments are listed in Figure S3. For the Sobs richness index and Shannon-Weaver diversity index, there were no significant differences among all treatments (*p* > 0.05), except for that the richness was significantly lower in NPK than in CK and M (*p* < 0.05). As for the Pielou evenness index, compared with UL, no significant differences were found in all fertilization treatments (*p* > 0.05), while there was a significant increase in NPK compared to in CK (*p* < 0.05).

### 3.3. Bacterial Community Composition, Biomarkers and Phenotypes

**Figure 1:**
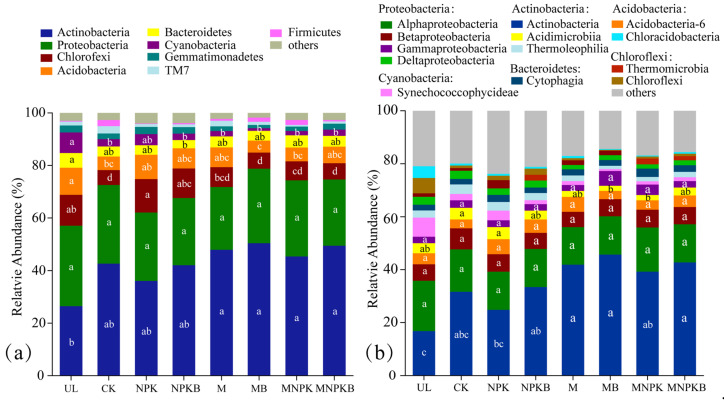
Soil bacterial community composition under different treatments at phylum (**a**) and class (**b**) level. Different lowercase letters within each taxa mean significant differences (*p* < 0.05) of abundance among treatments revealed by one-way ANOVA. UL, uncultivated land; CK, control without fertilization; NPK, chemical fertilizer; NPKB, co-application of chemical fertilizer and bacterial fertilizer; M, manure; MB, co-application of manure and bacterial fertilizer; MNPK, co-application of chemical fertilizer and manure; MNPKB, co-application of chemical fertilizer, manure, and bacterial fertilizer.

Figure 1 depicts the soil bacterial community composition of different treatments, and it can be seen that the dominant bacterial phyla among all treatments were *Actinobacteria* (26.44–50.42), *Proteobacteria* (23.87–30.64%), *Chloroflexi* (5.65–12.75%), *Acidobacteria* (4.58–10.35%), *Bacteroidetes* (3.13–5.58), *Cyanobacteria* (1.04–7.83%), *Gemmatimonadetes* (1.22–2.74%), *TM7* (0.61–2.80%), and *Firmicutes* (0.22–2.30%), accounting for 95.97–98.23% of all taxa (Figure 1a). One-way ANOVA indicated that all manure treatments (M, MB, MNPK, and MNPKB) significantly increased the abundance of *Actinobacteria* (*p* < 0.05) over UL, while they and CK significantly decreased that of *Chloroflexi* (*p* < 0.05). *Acidobacteria* and *Bacteroidetes* decreased among all reclamation treatments apart from UL, with significant differences in CK, MB, and MNPK for the former and only in NPKB for the latter (*p* < 0.05). Meanwhile, other than in NPK, *Cyanobacteria* was significantly decreased in all reclamation treatments other than UL (*p* < 0.05).

The dominant bacterial classes among all treatments were *Actinobacteria* (45.70–16.82%), *Alphaproteobacteria* (18.98–14.17%), *Betaproteobacteria* (7.97–5.76%), *Acidobacteria*-6 (5.65–3.18%), *Acidimicrobiia* (4.58–1.91%), *Gammaproteobacteria* (5.58–2.30%), *Synechococcophycideae* (7.26–0.77%), *Thermoleophilia* (3.61–1.20%), *Cytophagia* (3.14–2.06%), *Deltaproteobacteria* (3.08–1.62%), *Thermomicrobia* (3.10–1.02%), *Chloroflexi* (0.32–5.75%), and *Chloracidobacteria* (0.33–4.49%), accounting for 76.22–85.58% of all taxa (Figure 1b). In detail, the *Actinobacteria* class was increased in all reclamation treatments apart from UL, with significant differences in all fertilization treatments except for in NPK (*p* < 0.05). The abundance of *Acidimicrobiia* was significantly lower in MB and MNPK than in CK and NPK (*p* < 0.05). However, other classes showed no significant differences among treatments (*p* > 0.05).

**Figure 2:**
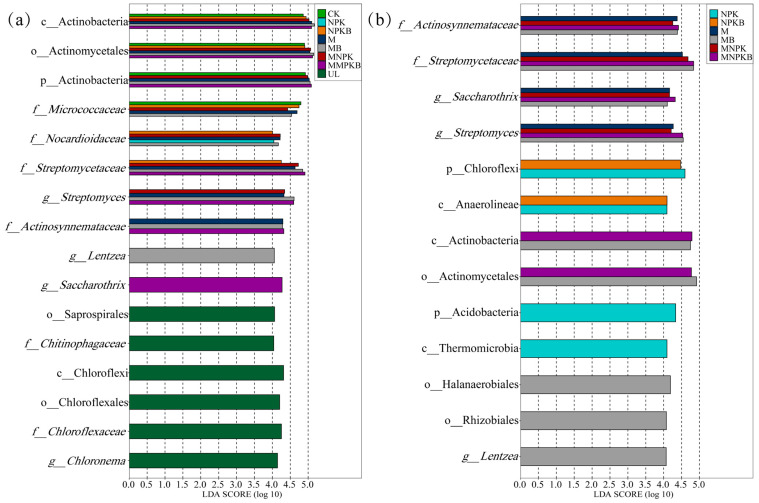
Phylotypic biomarkers determined by LEfSe algorithm (**a**) between UL and reclamation treatments (CK, NPK, NPKB, M, MB, MNPK, and MNPKB) and (**b**) between CK and fertilization treatments (NPK, NPKB, M, MB, MNPK, and MNPKB). The biomarkers in each reclamation or fertilization treatment were defined as those significantly enriched only compared to UL or CK, while the biomarkers of UL and CK were those significantly enriched compared to all reclamation or fertilization treatments. A significance level of *p* < 0.05 and a threshold of LDA > 4.0 were used for all the biomarkers. The lowercase prefix letters indicate the taxonomic level of the biomarker: p = phylum; c = class; o = order; f = family; g = genus. UL, uncultivated land; CK, control without fertilization; NPK, chemical fertilizer; NPKB, co-application of chemical fertilizer and bacterial fertilizer; M, manure; MB, co-application of manure and bacterial fertilizer; MNPK, co-application of chemical fertilizer and manure; MNPKB, co-application of chemical fertilizer, manure, and bacterial fertilizer.

LEfSe was used to figure out the phylotypic biomarkers that were significantly enriched (*p* < 0.05, the same below) among different treatments. As shown in Figure 2a, generally 16 biomarkers with an LDA > 4.0 were screened out, 10 of which belonged to *Actinobacteria* and were significantly enriched in reclamation treatments, while the other 6 belonged to *Chloroflexi* and were significantly enriched in UL. In detail, compared with UL, at phylum level, *Actinobacteria* was significantly enriched in CK and manure treatments (M, MB, MNPK, and MNPKB). At class and order level, *Actinobacteria* and *Actinomycetales* were, respectively, significantly enriched in all reclamation treatments other than NPK. *Streptomycetaceae* and *Streptomyces* were significantly enriched in manure treatments. In addition, M and MB also both significantly enriched *Micrococcaceae*, *Nocardioidaceae*, and *Actinosynnemataceae*. Compared with all seven of the reclamation treatments, *Saprospirales*, *Chitinophagaceae*, *Chloroflexi*, *Chloroflexales*, *Chloroflexaceae*, and *Chloronema* were significantly enriched in UL.

According to Figure 2b, 13 biomarkers with an LDA > 4.0 were found, 7 of which belong to *Actinobacteria* and 3 of which belong to *Chloroflexi*. Hence, fertilization mainly influenced the community formation of *Actinobacteria* and *Chloroflexi*. Compared with CK, *Actinosynnemataceae*, *Streptomycetaceae*, *Saccharothrix*, and *Streptomyces* were significantly enriched in manure treatments. *Chloroflexi* and *Anaerolineae* were significantly enriched in NPK and NPKB treatments. *Actinobacteria* and *Actinomycetales* were significantly enriched in manure treatments and bacterial fertilizer (MB and MNPKB) treatments. Compared with all six of the fertilization treatments, CK significantly enriched *Halanaerobiales*, *Rhizobiales*, and *Lentzea*.

**Figure 3:**
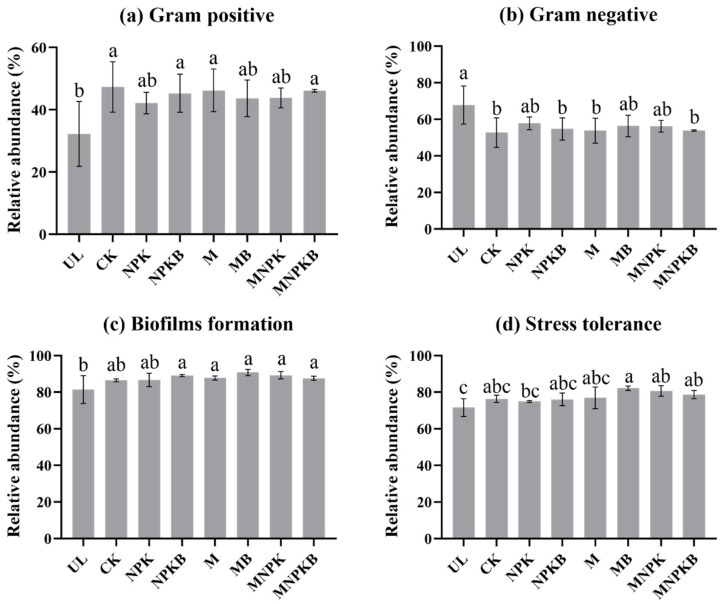
Relative abundance of soil bacterial phenotypic biomarkers with significant variations (*p* < 0.05) predicted by the BugBase algorithm. Different lowercase letters above each column mean significant differences (*p* < 0.05) of relative abundance among treatments revealed by one-way ANOVA. UL, uncultivated land; CK, control without fertilization; NPK, chemical fertilizer; NPKB, co-application of chemical fertilizer and bacterial fertilizer; M, manure; MB, co-application of manure and bacterial fertilizer; MNPK, co-application of chemical fertilizer and manure; MNPKB, co-application of chemical fertilizer, manure, and bacterial fertilizer.

Based on the BugBase algorithm, nine bacterial phenotypes, including facultative anaerobe, anaerobe, biofilms formation, contains mobile elements, Gram-positive, Gram-negative, potential pathogen, and stress tolerance, were predicted in the coal-mining soils. Among them, the relative abundances of Gram-positive, Gram-negative, biofilms formation, and stress tolerance were found to be significantly different (*p* < 0.05) among treatments (Figure 3). In detail, compared with UL, the relative abundance of Gram-positive bacteria increased (Figure 3a) while that of Gram-negative bacteria decreased (Figure 3b) after reclamation, which reached a significant level in CK, NPKB, M, and MNPKB (*p* < 0.05). For biofilms-forming bacteria, the abundance increased after reclamation, and significant differences were found in all fertilization treatments except for NPK (Figure 3c). An increase after reclamation was also observed in the relative abundance of stress-tolerant bacteria, which was significant in MB, MNPK, and MNPKB (Figure 3d).

### 3.4. Bacterial Community Structure Differentiations and Driving Factors Among Different Treatments

**Figure 4:**
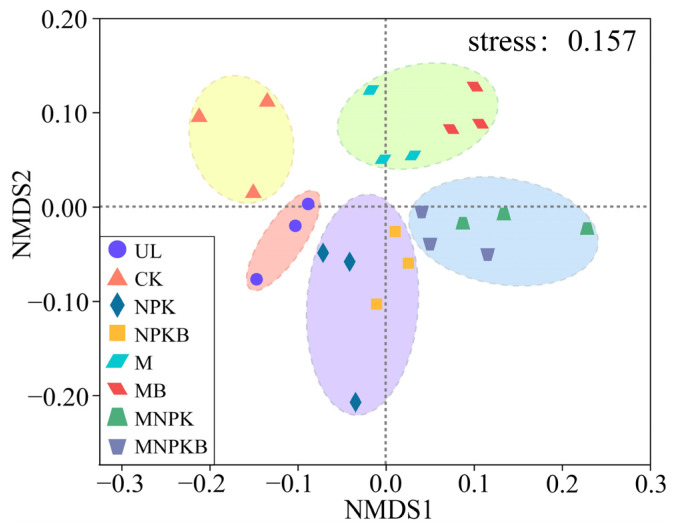
Non-metric multidimensional scaling (NMDS) of bacterial community structures among treatments at genera level. UL, uncultivated land; CK, control without fertilization; NPK, chemical fertilizer; NPKB, co-application of chemical fertilizer and bacterial fertilizer; M, manure; MB, co-application of manure and bacterial fertilizer; MNPK, co-application of chemical fertilizer and manure; MNPKB, co-application of chemical fertilizer, manure, and bacterial fertilizer.

NMDS and a biclustering heatmap were applied to investigate the bacterial community structures among treatments. As shown in Figure 4, the NMDS biplot showed a good representation of the distribution of bacterial communities (stress < 0.2, *p* < 0.05), revealing that they grouped differently according to reclamation and fertilization. The eight treatments fell into five clusters according to their bacterial genera composition, with good consistency within each treatment. Each fertilization treatment pairs with/without bacterial fertilizer (NPK vs. NPKB, M vs. MB, MNPK vs. MNPK) harbored similar community structures, clustered together and separated away from UL and CK. Among them, manure treatments (M, MB, MNPK, and MNPKB) distributed further away from UL and CK. Furthermore, the biclustering heatmap showed a similar differentiation pattern among treatments and a more obvious successive variation from UL to CK and then to fertilization treatments (Figure S4).

**Figure 5:**
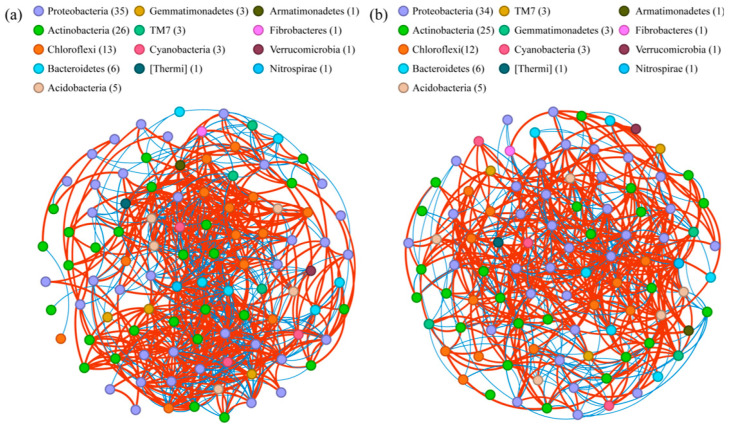
Correlation networks of bacterial communities in the (**a**) no-manure group and (**b**) manure group. The no-manure group includes UL, CK, NPK, and NPKB, while the manure group includes M, MB, MNPK, and MNPKB. Both of the networks are constructed based on the robust significant Spearman’s correlation effects (|*R*| > 0.60, *p* < 0.01). Each node, respectively, represents a bacterial genus, whose color indicates its phylum affiliation according to the legend. The digit in each bracket in the legend shows the number of genera of the corresponding phylum that are included in the network. Each edge represents a correlation effect, which indicates a positive correlation with red color and a negative correlation with blue.

According to the general differentiation pattern of bacterial community structures identically revealed by NMDS and the biclustering heatmap, the eight treatments were classified into two groups, the manure group (M, MB, MNPK, and MNPKB) and the no-manure group (UL, CK, NPK, and NPKB), to construct Spearman’s correlation networks based on the top 100 bacterial genera composition, respectively. The no-manure group (Figure 5a) harbored a more complex network than the manure group (Figure 5b), which could also be implied by the topological parameters of the networks (Table S2).

**Figure 6:**
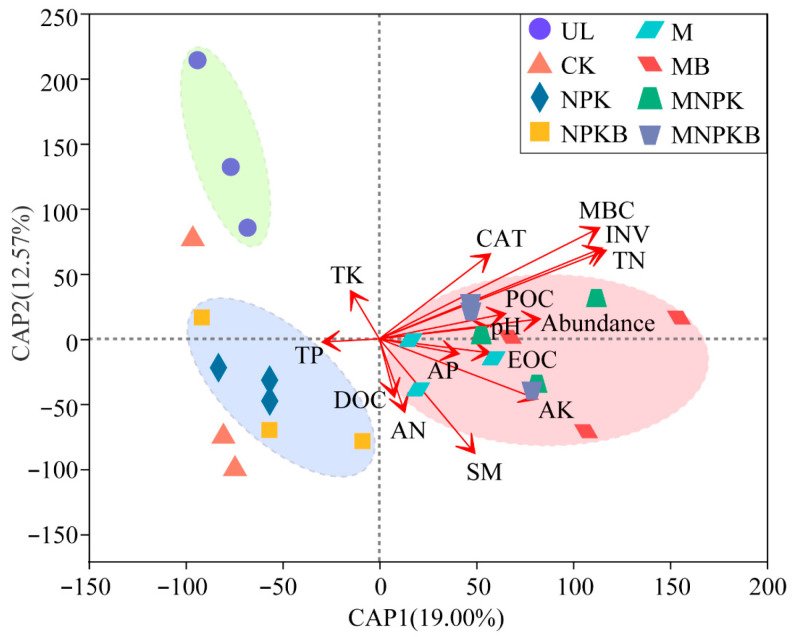
Distance-based redundancy analysis (db-RDA) between bacterial genera composition and environmental variables. SM, soil moisture; TN, total nitrogen; AN, alkaline nitrogen; TP, total phosphorus; AP, available phosphorus; TK, total potassium; AK, available potassium; MBC, microbial biomass carbon; EOC, easily oxidized organic carbon; DOC, dissolved organic carbon; POC, particulate organic carbon; CAT, catalase; INV, invertase. UL, uncultivated land; CK, control without fertilization; NPK, chemical fertilizer; NPKB, co-application of chemical fertilizer and bacterial fertilizer; M, manure; MB, co-application of manure and bacterial fertilizer; MNPK, co-application of chemical fertilizer and manure; MNPKB, co-application of chemical fertilizer, manure, and bacterial fertilizer.

Distance-based redundancy analysis (db-RDA) was conducted to further reveal the driving factors of the above bacterial community structure differentiations, with soil biophysicochemical properties as environmental variables. The variables with a VIF > 5 were eliminated before db-RDA to avoid multicollinearity. As Figure 6 depicts, the first two axes totally explained 31.57% of the bacterial community structure variations among treatments. The eight treatments generally were separated into two clusters, the manure treatments and the no-manure treatments. It was consistent with NMDS and the biclustering heatmap. The main driving factors exerting the differentiation of fertilization treatments from UL were SM (*r* = 0.25, *p* = 0.038) and AK (*r* = 0.38, *p* = 0.05). While key factors differentiating manure treatments from no-manure treatments were MBC (*r* = 0.70, *p* = 0.001), TN (*r* = 0.62, *p* = 0.001), INV (*r* = 0.61, *p* = 0.001), and CAT (*r* = 0.26, *p* = 0.048).

**Figure 7:**
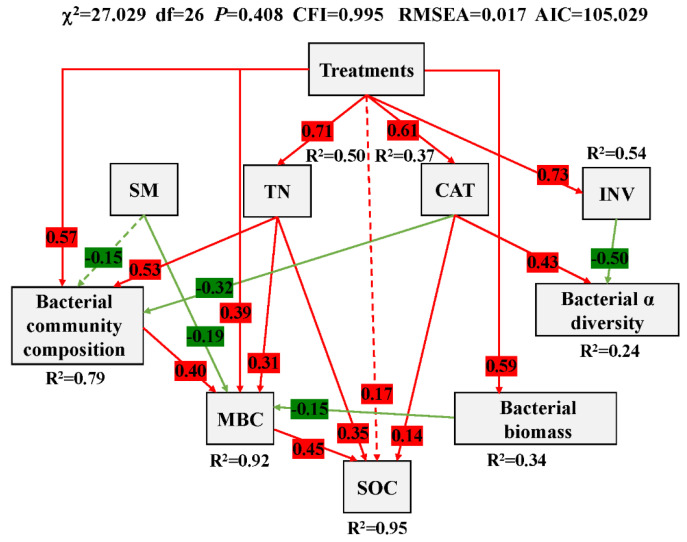
Structural equation model (SEM). It shows the causal influences of treatments (manure application dose), SM (soil moisture), TN (total nitrogen), CAT (catalase), INV (invertase), bacterial α diversity (Shannon-Weaver index), bacterial community composition (loading scores on the NMDS axis 1), bacterial biomass (16S rRNA gene abundance), SOC (soil organic carbon), and MBC (microbial biomass carbon). Positive and negative effects are, respectively, shown in red and green, and significant and non-significant effects are shown with solid and dashed arrow lines, respectively. The standardized coefficients are marked above each path (only significant effect paths are marked) and indicate the expected amount of change at one node per one standard deviation change in the connected nodes. R^2^ values represent the proportion of the variance explained for each endogenous variable.

Subsequently, the structural equation model (SEM) was employed to elucidate the detailed interactions of soil bacterial communities, the driving factors, and the reclamation treatments (Figure 7). According to the fit indices (χ^2^/df < 2, *p* > 0.05, GFI > 0.95, RMSEA < 0.05), the model provided an adequate fitness to the data, which could, respectively, explain 95%, 92%, 79%, 34%, and 24% variance of the SOC, MBC, bacterial community composition, biomass, and α-diversity. The SOC was directly influenced by the MBC, TN and CAT, as well as indirectly influenced by the bacterial community composition (standard coefficient = 0.177), bacterial biomass (standard coefficient = −0.066), and SM (standard coefficient = −0.11). The MBC was directly influenced by the treatments (manure application dose), TN, SM, bacterial community composition, and biomass, as well as indirectly influenced by the CAT (standard coefficient = −0.128). The bacterial community composition was directly influenced by the treatments, TN and CAT. The bacterial biomass was directly influenced by the treatments. The bacterial α diversity was directly influenced by the CAT and INV.

## 4. Discussion

Fertilization is an appropriate management measure to recover soil productivity and quality [29]. In this study, we investigated the impact of different fertilization practices on bacterial diversity and community structure and identified the key driving factors in coal-mining reclaimed soils. The results demonstrated that all the six fertilization practices significantly increased the soil organic carbon, nitrogen and phosphorus nutrients (TN and AP), microbial biomass (MBC), and nutrients cycling enzymes activities (ALP, URE, CAT, and INV). The bacterial community structure was significantly differentiated under different treatments and several significantly enriched phylotypic and phenotypic bacterial biomarkers were unveiled. Soil properties, particularly the total nitrogen (TN), microbial biomass carbon (MBC), and enzyme activities such as invertase (INV) and catalase (CAT), emerged as the key driving factors of bacterial community structure. These findings highlight the critical role of soil amendments in modulating microbial communities and soil health at the initial stage of coal-mining soil reclamation.

Although the MBC was significantly increased in all fertilization treatments, bacterial biomass represented by the abundance of the 16S rRNA gene was only significantly increased after the co-application of manure and bacterial fertilizer (MB). The biomass of other microorganisms, such as fungi and protists, was more sensitive to reclamation and fertilization than bacteria, especially in such a short term [30,31]. Nevertheless, reclamation, especially amendments of fertilizers, significantly altered the soil bacterial community structure (Figure 4, Figure 6 and Figure S4). All fertilization enhanced the soil urease (URE) and alkaline phosphatase (ALP), and consequently increased the soil total nitrogen (TN) and available phosphorus (AP). Notably, the enhancement was stronger in bacterial fertilizer co-application treatments (NPKB, MB, and MNPKB) than their corresponding non-co-application counterparts (NPK, M, and MNPK), respectively, as well as the MBC increase (Table 1). This short-term stimulation effect on the soil bacterial community structure and activity was due to the rapid dissolution and diffusion of fertilizers [32,33]. Moreover, the co-application of bacterial fertilizer further promoted the stimulation due to the “artificially” increased biomass of beneficial bacteria and their beneficial secretions, and the bifunction of nitrogen metabolism and phosphate-solubilizing provided by *Pseudomonas fluorescence* strains [20,34].

For in-depth detail on the bacterial community structure variation after 1-year reclamation, the bacterial phylotypic biomarkers were fertilizer-dependent as the phylum *Actinobacteria* was significantly enriched in manure treatments (M, MB, MNPK, and MNPKB) (Figure 2). Fierer et al. [35] proposed a copiotrophic hypothesis and predicted that copiotrophic bacteria harbor fast-growing rates and prefer higher organic carbon and nutrients, while oligotrophic bacteria harbor slow-growing rates and likely thrive in a low-nutrient environment. *Actinobacteria* were identified as copiotrophs and could be enriched after manure fertilization, so its relative abundance significantly increased in all manure fertilization treatments and reached up to a one-fold increase in MB [36,37]. For the enrichment of *Actinobacteria* in CK (without fertilization) compared to UL, it could be attributed to the input of labile organic carbon that maize root exudates [38]. Bacterial biomarkers in all manure treatments also included *Streptomycetaceae* and *Actinosynnemataceae*, two prolific antibiotic-producing families, and their dominant genus *Streptomyces* and *Saccharothrix* [39,40]. These taxa were either non-inhibited or resistant to possible antibiotics that might be introduced into the soil via manure fertilization and were possibly enriched at the expense of a decreased relative abundance of other bacteria [41,42,43,44]. Nevertheless, manure fertilization, other than chemical fertilizer, could enrich resident antibiotic-resistant bacteria in soil even though the animals from which the manure was derived had not been treated with antibiotics [45].

For chemical fertilizer treatments (NPK and NPKB), the enriched biomarkers were oligotrophic phylum *Chloroflexi* and its class *Anaerolineae*, which were also found to be significantly induced in chemically fertilized soils [46]. Furthermore, chemical fertilizer was found to induce oligotrophic *Acidobacteria*, whose enrichment in NPK was also observed in this study (Figure 2) by changes in soil pH [46]. Here, we predict that *Chloroflexi* are also induced by pH variations resulting from the application of chemical fertilization, as a pH decreasing trend in NPK and NPKB compared to other treatments is observed (Table 1) and the correlation between *Chloroflexi* and soil pH has been previously reported [47,48].

As for the predicted phenotypic biomarkers, the relative abundance of Gram-positive bacteria increased in all reclamation treatments (CK and fertilization treatments), compared to UL, while Gram-positive bacteria decreased (Figure 3). It was consistent with the phylotypic biomarkers, as the Gram-positive *Actinobacteria* and its taxa [49], from phylum to genus level, were significantly enriched in all reclamation treatments. The increase in biofilms-formation phenotypes in all reclamation treatments might be attributed to the higher nutrients’ availability from fertilizers as well as the maize root exudates, as shown in Table 1 [50]. The enrichment of stress tolerance might be due to the oxidative stress reducing by catalase [51] and the antibiotics inhibition or tolerance by the enriched *Actinobacteria* mentioned above.

The counterintuitive less complex co-occurrence network of the manure group (M, MB, MNPK, and MNPKB) compared to that of the no-manure group (UL, CK, NPK and, NPKB), in fact, further implied that the manure application enhanced the bacterial community. As previously revealed, the complexity of a bacterial phylotype network decreased in more productive soils [52]. The harsher nutrients deficiency in the no-manure group (Table 1) stimulated the effect of environmental filtering and exerted more coherent bacterial functional traits and increased intraspecific interactions, characterized by a more complex co-occurrence network. While for the manure group, it was quite the opposite [53].

As revealed by distance-based redundancy analysis (db-RDA) (Figure 6), the bacterial community structures exhibited significant differences among various treatments, primarily influenced by the application of manure. It was also corroborated by the structural equation model (SEM) where the composition, diversity, and biomass of bacterial communities were directly or indirectly affected by treatments, which were characterized by differing manure application doses (Figure 7). Collectively, for the driving factors of bacterial community, it was implied that during the one-year reclamation, fertilization, especially manure amendments, had boosted nutrients supply (SOC, TN, and AK) and supported microbial growth (MBC) and metabolic diversity (CAT and INV) [54]. Activity of invertase, essential for breaking down simple carbohydrates to provide an energy source for soil microorganisms [55], was an effective indicator for monitoring and assessing soil-rehabilitation processes in industrial areas [56]. Its activity increased due to the availability of organic substrates from fertilization (Table 1). This enhancement facilitated more efficient carbon cycling and exerted positive feedback within the soil system for reclamation. Catalase, vital for reducing oxidative stress and reflecting soil aeration and microbial metabolic activity, was considered to be a good biological indicator of soil fertility [57,58]. Its activity was sustained in manure treatments rather than chemical fertilizer, which further implied the conduciveness of manure fertilizer in coal-mining soil reclamation.

Coal-mining soils typically lack essential nutrients and have a limited capacity to retain water [59]. This issue is particularly pronounced in regions like the Loess Plateau, where the soil environment is inherently fragile [60]. Consequently, even minor fluctuations in nutrient availability and soil moisture can provoke substantial changes within microbial communities. Such sensitivity underscores the critical role of fertilizer application in managing these ecosystems effectively, even after just one-year of reclamation. Microbial recovery in degraded coal-mining soil is estimated to occur within 5–14 years of reclamation [17]. Therefore, previous studies on microbial communities in subsiding coal-mining soils have primarily examined recovery over periods of 5 years or longer [13,14,16]. Some researchers included shorter terms in their reclamation chronosequence studies, but unfortunately not enough attention was paid on them [6,61,62,63,64]. Fewer studies focus on the bacterial community variation after one-year of reclamation and fertilization in coal-mining areas. Nevertheless, the neglected one-year short-term coal-mining reclamation data still silently indicated an obvious enhancement of bacterial diversity and community structure, which is consistent with our study, but without the detailed exploration we performed [6,11,12].

On the other hand, the reclamation of barren coal-mining soils is actually similar to the pedogenesis of a young soil or parent material [65]. Li et al. [33] reported that short-term organic fertilizer application within 1 year could significantly increase the bacterial abundance and alter the bacterial community structure in the soil parent material in the Karst region. The consistency with our study implied that organic fertilizer application is a promising agricultural fertilization in fragile ecosystems [33]. Our study demonstrated that one-year short-term reclamation, especially manure application, could elevate microbial biomass carbon, nutrients level, and enzyme activities. These shifts create diverse niches, revitalize barren soils, foster bacterial diversity, and alter their community structure from oligotrophic to copiotrophic. Notably, the priming effect induced by organic fertilization persists beyond the initial recovery phase, as evidenced by the sustained proliferation of copiotrophic taxa observed seven years post-reclamation [6]. This longitudinal perspective reveals that organic amendments not only initiate microbial community restructuring but also establish persistent ecological trajectories favoring nutrient-adapted bacterial consortia. The study establishes a mechanistic framework linking short-term biogeochemical stimulation with long-term microbial succession patterns, providing critical theoretical foundations for optimizing organic fertilization strategies in post-mining ecosystem restoration.

## 5. Conclusions

Our study revealed that even just one-year short-term reclamation with manure amendments could significantly enhance the soil microbial biomass, SOC, nutrients (TN and AP) and enzymes activities in coal-mining areas. The bacterial communities structures in manure-amended treatments were phylotypically shifted from oligotrophic to *Actinobacteria*-dominant copiotrophic traits, accompanied with the phenotypic succession of the enriching characteristics of Gram-positive, biofilms formation, and stress tolerance. Manure-amended treatments harbored a simple co-occurrence network, indicating more productive soils, than no-manure treatments. The key driving factors were manure amendments, MBC, TN, invertase, catalase, and soil moisture. Our study unveiled the bacterial initialization characteristics of soil ecology recovery at the very beginning of coal-mining reclamation with manure amendments. Future investigations should prioritize elucidating the ecological functional diversity and metabolic activity of those pioneer bacterial communities during the early-stage of coal-mining soil reclamation, with particular emphasis on their carbon sequestration capability.

## Figures and Tables

**Table 1:** Soil biophysicochemical properties in different treatments.

	Treatment							
	UL	CK	NPK	NPKB	M	MB	MNPK	MNPKB
SM	0.17 ± 0.02 abc	0.19 ± 0.02 a	0.15 ± 0.01 bcd	0.15 ± 0.01 cd	0.18 ± 0.01 ab	0.19 ± 0.01 a	0.15 ± 0.01 cd	0.14 ± 0.01 d
pH	8.27 ± 0.09 a	7.86 ± 0.49 a	7.85 ± 0.42 a	8.09 ± 0.04 a	8.15 ± 0.06 a	8.10 ± 0.03 a	8.14 ± 0.13 a	8.01 ± 0.28 a
TN (g/kg)	0.27 ± 0.04 d	0.32 ± 0.06 cd	0.41 ± 0.08 c	0.57 ± 0.06 b	0.56 ± 0.01 b	0.77 ± 0.03 a	0.59 ± 0.06 b	0.62 ± 0.06 b
AN (mg/kg)	304.05 ± 0.001 ab	344.59 ± 11.50 a	302.85 ± 30.84 ab	151.43 ± 52.37 c	315.97 ± 145.67 a	331.47 ± 69.02 a	174.08 ± 99.02 bc	162.16 ± 38.25 c
TP (g/kg)	0.38 ± 0.001 a	0.69 ± 0.46 a	0.39 ± 0.20 a	0.34 ± 0.19 a	0.35 ± 0.28 a	0.52 ± 0.18 a	0.39 ± 0.20 a	0.33 ± 0.24 a
AP (g/kg	5.09 ± 0.01 d	10.93 ± 0.26 bc	17.68 ± 5.80 a	12.72 ± 1.88 ab	12.14 ± 0.80 ab	12.08 ± 1.50 ab	18.65 ± 7.16 a	9.59 ± 2.03 bc
TK (g/kg)	5.61 ± 0.02 a	3.72 ± 0.74 bc	2.88 ± 1.40 c	4.10 ± 0.66 bc	3.20 ± 0.03 bc	4.45 ± 0.05 ab	4.01 ± 0.70 bc	4.03 ± 0.64 bc
AK (mg/kg)	80.06 ± 9.21 ab	74.71 ± 8.34 b	77.39 ± 8.34 b	70.71 ± 6.12 b	86.73 ± 10.08 ab	98.75 ± 18.51 a	86.74 ± 15.17 ab	72.05 ± 6.94 b
SOC (g/kg)	1.74 ± 0.03 e	1.55 ± 0.08 e	2.07 ± 0.17 d	2.62 ± 0.34 c	3.35 ± 0.24 ab	3.64 ± 0.16 a	3.12 ± 0.04 b	3.31 ± 0.21 ab
MBC (mg/kg)	9.85 ± 0.45 f	17.06 ± 1.50 e	19.72 ± 0.58 d	28.26 ± 1.88 c	37.78 ± 2.29 b	38.51 ± 0.99 ab	37.45 ± 0.82 b	40.75 ± 1.44 a
DOC (g/kg)	0.08 ± 0.01 a	0.09 ± 0.02 a	0.08 ± 0.01 a	0.07 ± 0.01 a	0.08 ± 0.01 a	0.09 ± 0.01 a	0.08 ± 0.01 a	0.08 ± 0.01 a
EOC (g/kg)	1.58 ± 0.22 a	1.48 ± 0.58 a	1.34 ± 0.35 a	1.51 ± 0.36 a	1.93 ± 0.46 a	1.54 ± 0.30 a	1.65 ± 0.69 a	1.47 ± 0.12 a
POC (g/kg)	0.24 ± 0.23 a	0.28 ± 0.15 a	0.52 ± 0.12 a	0.46 ± 0.07 a	0.60 ± 0.18 a	0.75 ± 0.36 a	0.71 ± 0.57 a	0.59 ± 0.03 a
ALP (μg/g)	1.88 ± 0.13 e	2.50 ± 1.07 e	5.11 ± 0.79 d	6.07 ± 0.70 cd	9.48 ± 0.43 b	11.21 ± 1.53 a	7.13 ± 0.19 c	9.11 ± 0.73 b
CAT (mL/g)	2.15 ± 0.06 a	1.52 ± 0.22 d	1.65 ± 0.03 cd	1.78 ± 0.03 bc	2.22 ± 0.05 a	2.10 ± 0.08 a	1.97 ± 0.26 ab	2.18 ± 0.10 a
INV (mg/g)	1.40 ± 0.12 cd	1.23 ± 0.16 d	1.35 ± 0.09 cd	1.54 ± 0.13 bc	1.66 ± 0.06 b	1.88 ± 0.06 a	1.53 ± 0.18 bc	1.66 ± 0.06 b
URE (mg/g)	0.11 ± 0.02 d	0.10 ± 0.01 d	0.22 ± 0.02 d	0.48 ± 0.06 c	0.98 ± 0.11 b	1.53 ± 0.27 a	0.99 ± 0.11 b	1.17 ± 0.18 b

Notes: SM, soil moisture; TN, total nitrogen; AN, alkaline nitrogen; TP, total phosphorus; AP, available phosphorus; TK, total potassium; AK, available potassium; SOC, soil organic carbon; MBC, microbial biomass carbon; EOC, easily oxidized organic carbon; DOC, dissolved organic carbon; POC, particulate organic carbon; ALP, alkaline phosphatase; URE, urease; CAT, catalase; INV, invertase. UL, uncultivated land; CK, control without fertilization; NPK, chemical fertilizer; NPKB, co-application of chemical fertilizer and bacterial fertilizer; M, manure; MB, co-application of manure and bacterial fertilizer; MNPK, co-application of chemical fertilizer and manure; MNPKB, co-application of chemical fertilizer, manure, and bacterial fertilizer. Different lowercase letters within each column mean significant differences (*p* < 0.05) among treatments revealed by one-way ANOVA. Below is the same.

## Data Availability

The raw sequencing data obtained from this study were submitted to the National Center for Biotechnology Information Sequence Read Archive (SRA) and the BioProject under the following ID: PRJNA948914.

## Supplementary Materials

The following supporting information can be downloaded at: https://www.mdpi.com/article/10.3390/microorganisms13040699/s1, Figure S1: Schematic diagram of the geographic location of the study site; Figure S2: Abundance of soil 16S rRNA gene under different treatments; Figure S3: Soil bacterial alpha diversity indexes under different treatments; Figure S4: Biclustering heatmap of the top 35 abundant bacterial genera among different treatments; Table S1: Fertilization dose in different treatments (kg/hectare); Table S2: Topological parameters of the correlation networks.

## Abbreviations

The following abbreviations are used in this manuscript:

SM	Soil moisture
TN	Total nitrogen
AN	Alkaline nitrogen
TP	Total phosphorus
AP	Available phosphorus
TK	Total potassium
AK	Available potassium
SOC	Soil organic carbon
MBC	Microbial biomass carbon
EOC	Easily oxidized organic carbon
DOC	Dissolved organic carbon
POC	Particulate organic carbon
ALP	Alkaline phosphatase
URE	Urease
CAT	Catalase
INV	Invertase
UL	Uncultivated land
CK	Control without fertilization
NPK	Chemical fertilizer
NPKB	Co-application of chemical fertilizer and bacterial fertilizer
M	Manure
MB	Co-application of manure and bacterial fertilizer
MNPK	Co-application of chemical fertilizer and manure
MNPKB	Co-application of chemical fertilizer manure and bacterial fertilizer
