# Supplementary material for: Manure-Amended One-Year-Reclamation Promoted Soil Bacterial Phylotypic and Phenotypic Shifts in a Typical Coal-Mining Area
Source: Microorganisms. 2025 Mar 21;13(4):699. doi: 10.3390/microorganisms13040699 (PMC12029533; doi:10.3390/microorganisms13040699)
Supplement: Supplementary file 1 [file microorganisms-13-00699-s001.zip › microorganisms-3531623-supplementary.pdf]

Table S1. Fertilization dose in different treatments (kg/hectare).

**Treatments**	**Urea (N, 46%)**	**Calcium superphosphate (P_2O_5, 16%)**	**Potassium chloride (K_2O, 60%)**	**Manure**	**phosphate solubilizing bacterial fertilizer**
UL	0	0	0	0	0
CK	0	0	0	0	0
NPK	653	1384	324	0	0
NPKB	599	1268	297	0	1500
M	0	0	0	18000	0
MB	0	0	0	16500	1500
MNPK	326.5	692	162	9000	0
MNPKB	326.5	692	162	7500	1500

Table S2. Topological parameters of the correlation networks.

**Treatments**	**No-manure group (UL, CK, NPK, NPKB)**	**Manure group (M, MB, MNPK, MNPKB)**
Number of Nodes	99	96
Total number of Edge (correlation effects)	680	541
Number of positive correlation effects	416	333
Number of negative correlation effects	264	208
Network diameter	8	5
Graph density	0.14	0.119
Modularity	1.727	1.955
Average path length	2.627	2.435

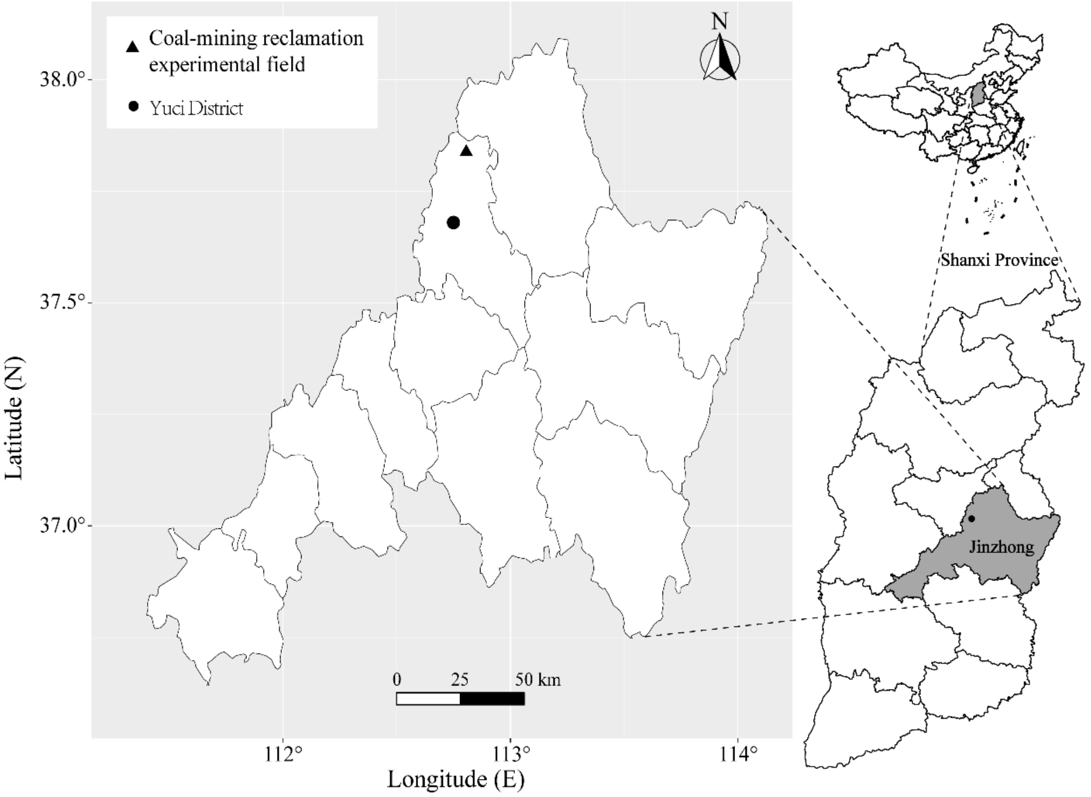

Figure S1 Schematic diagram of the geographic location of the study site

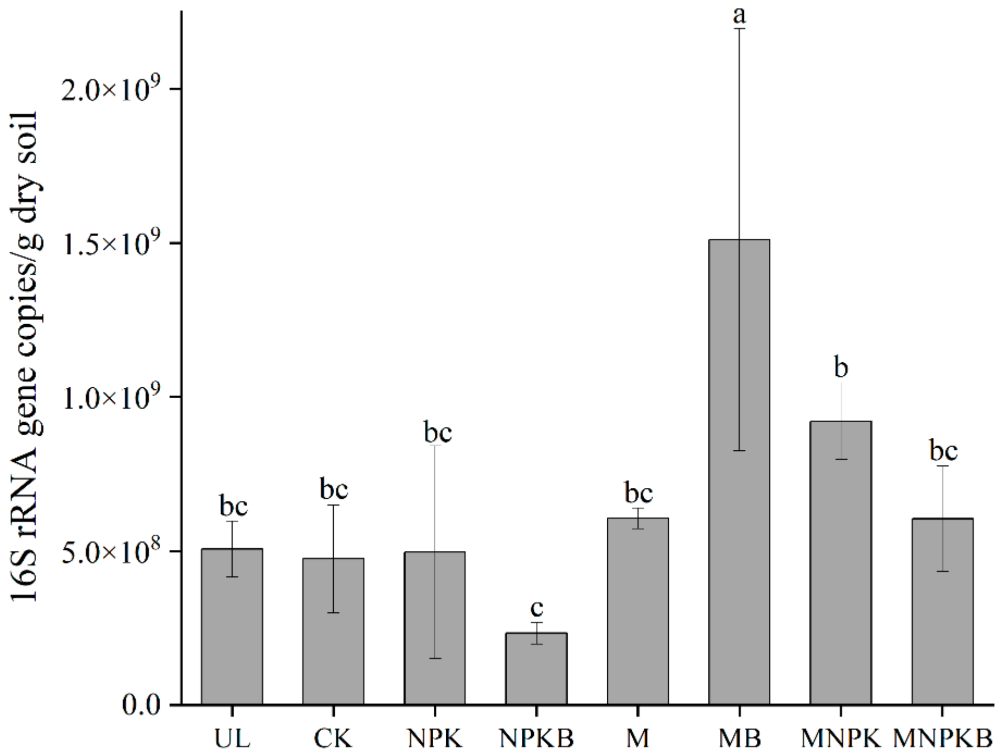

Figure S2. Abundance of soil 16S rRNA gene under different treatments.

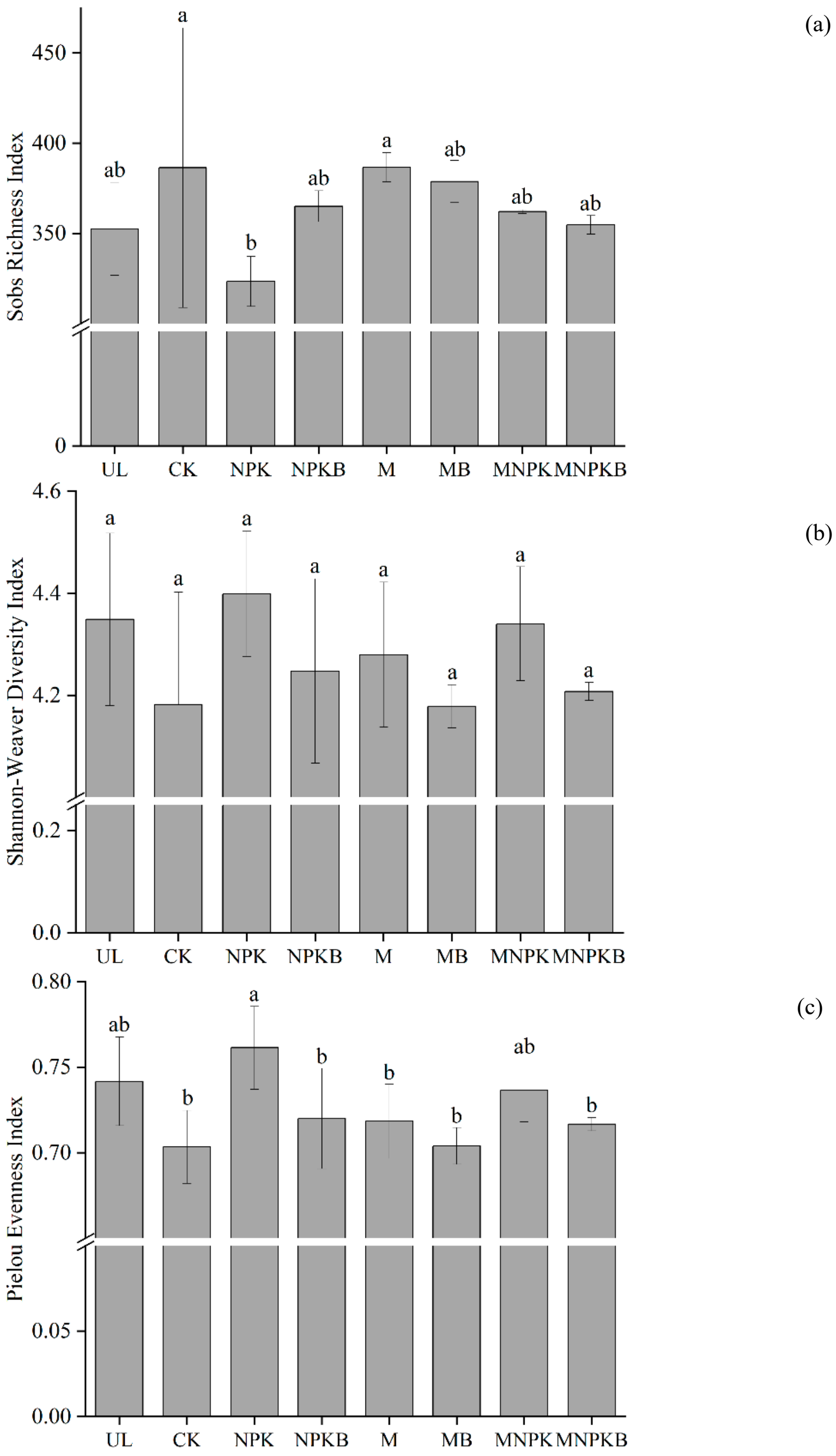

Figure S3 Soil bacterial alpha diversity indexes under different treatments.

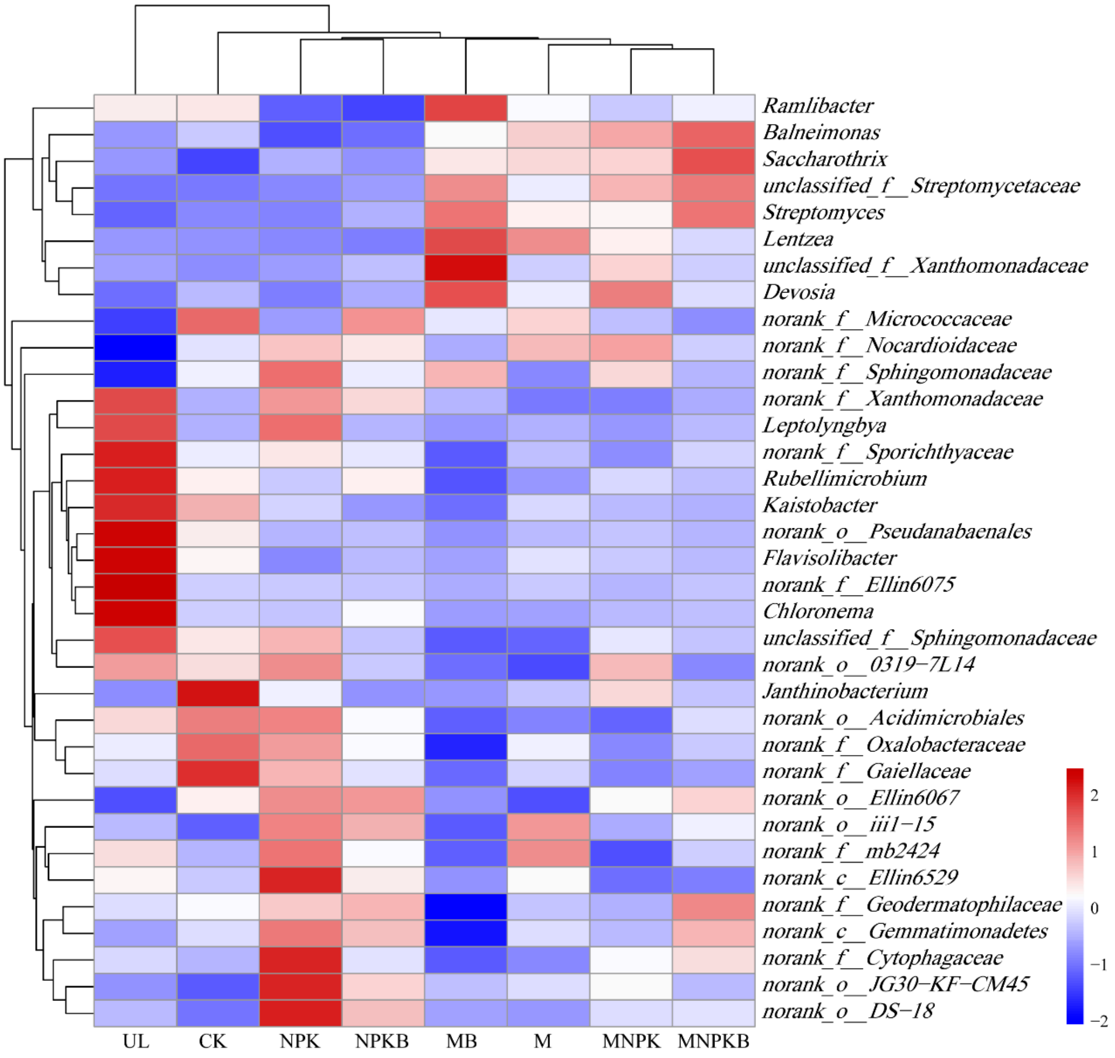

.

Figure S4. Biclustering heatmap of the top 35 abundant bacterial genera among different treatments. The cell color intensity represents the relative abundance of bacterial genera, with red representing higher abundance and blue lower.
